# Processing and Characterization of High-Density Fe-Silicide/Si Core–Shell Quantum Dots for Light Emission

**DOI:** 10.3390/nano15100733

**Published:** 2025-05-14

**Authors:** Katsunori Makihara, Yuji Yamamoto, Markus Andreas Schubert, Andreas Mai, Seiichi Miyazaki

**Affiliations:** 1 Graduate School of Engineering, Nagoya University, Nagoya 464-8601, Japan; yamamoto@ihp-microelectronics.com; 2 IHP-Leibniz-Institut für Innovative Mikroelektronik, 15236 Frankfurt, Germany; schuberta@ihp-microelectronics.com (M.A.S.); mai@ihp-microelectronics.com (A.M.); 3 Hiroshima University, Higashihiroshima 739-0046, Japan; semiya1@hiroshima-u.ac.jp

**Keywords:** Fe-silicide, core/shell structure, CVD

## Abstract

Si-based photonics has garnered considerable attention as a future device for complementary metal–oxide–semiconductor (CMOS) computing. However, few studies have investigated Si-based light sources highly compatible with Si ultra large-scale integration processing. In this study, we observed stable light emission at room temperature from superatom-like β–FeSi_2_–core/Si–shell quantum dots (QDs). The β–FeSi_2_–core/Si–shell QDs, with an areal density as high as ~10^11^ cm^−2^ were fabricated by self-aligned silicide process of Fe–silicide capped Si–QDs on ~3.0 nm SiO_2_/n–Si (100) substrates, followed by SiH_4_ exposure at 400 °C. From the room temperature photoluminescence characteristics, β–FeSi_2_ core/Si–shell QDs can be regarded as active elements in optical applications because they offer the advantages of photonic signal processing capabilities and can be combined with electronic logic control and data storage.

## 1. Introduction

The monolithic integration of Si-based photonics with electronic processes on a single chip is considered a key advancement for beyond–complementary metal–oxide–semiconductor (CMOS) computing [1,2,3,4,5,6,7,8,9]. Photonic components utilize photons allowing for the highest level of precision and highly efficient data processing and transmission, resulting in faster and more energy-efficient devices. However, from an application point of view, Si-based photonics necessitate Si-based light sources that are highly compatible with Si–ultra-large-scale integration (Si–ULSI) processing [10,11,12,13,14,15,16,17,18,19,20,21,22,23,24,25,26,27,28,29,30,31,32,33,34]. Most importantly, despite the excellent optical properties of Si-based light emitters due to their high refractive index and the high-quality Si/SiO_2_ interface, which means low defect density, they suffer from the inherent indirect band gap of Si, resulting in long carrier lifetimes and inefficient light emission. Therefore, integration of Si-based photonic processing with electrical processing on a single chip remains a significant challenge. In this study, we report on light emission from Si quantum dots (QDs) with a β–FeSi_2_ core referred to as superatom-like QDs. β–FeSi_2_ is a semiconductor phase with an indirect bandgap of ~0.7 eV, close to the direct bandgap value (~0.85 eV) at room temperature and shows light emission in the near-infrared region [35,36,37,38,39,40,41,42,43]. Therefore, to achieve good thermal stability and light emission characteristics, β–FeSi_2_ is one of the promising candidates.

Si-based QDs have garnered significant attention due to their potential application as active elements in group IV photonics, driven by their light emission properties attributed to the carrier confinement effect. Previously, we reported the self-assembly of Si-QDs with an areal density as high as ~10^11^ cm^−2^ on ultrathin SiO_2_ by controlling the early stages of thermal decomposition of SiH_4_, showcasing their light emission properties [44,45,46]. More recently, we also detailed the formation of the β–FeSi_2_ nanodots (NDs) through the exposure of an ultrathin Fe layer to a remote H_2_ plasma (H_2_–RP) followed by SiH_4_ exposure, and characterized their photoluminescence (PL) properties [47,48,49]. In this work, we successfully formed high-density Si–shell/β–FeSi_2_–core QDs, akin to superatoms, with a uniform size distribution by controlling self-aligned silicidation of the Si–QDs and subsequent selective Si growth at low temperature to minimize metal diffusion. Furthermore, we discovered that light emission with a narrow wavelength range at room temperature from the Si–QD with a β–FeSi_2_ core was due to radiative recombination between the electron-quantized state in the core and the hole-quantized state in the shell, indicating that the Si–QD with a β–FeSi_2_–core is a promising light-emitting material for Si-based photonics.

## 2. Materials and Methods

**Figure 1:**
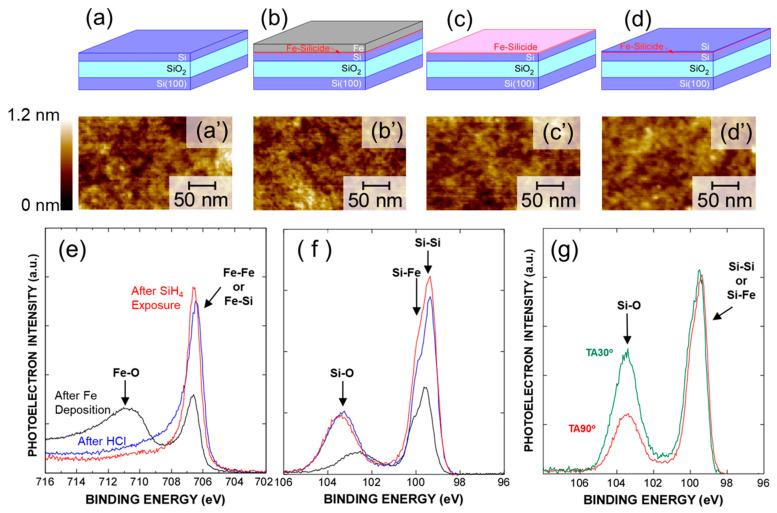
(**a**–**d**) Schematic illustration at each process step, and corresponding (**a′**–**d′**) typical AFM images of (**a**,**a′**) SOI surface, (**b**,**b′**) taken after Fe film deposition, (**c**,**c′**) after HCl treatment, and (**d**,**d′**) after subsequent SiH_4_ exposure. (**e**) Fe2p, and (**f**,**g**) Si2p core–line spectra of Fe/SOI substrate before and after HCl treatment, and after SiH_4_ exposure taken at photoelectron take-off angle of (**e**,**f**) 90° and (**g**) 30°.

To systematically investigate the silicide process and selective Si growth, Si on insulator (SOI) substrates was used in this study, with a SiO_2_ thickness of ~145 nm. Figure 1a–d schematically illustrates the concept of the experiment. Initially, the Si layer on the SiO_2_ was thinned to ~10 nm by alternately controlling the thermal oxidation and wet etching using a diluted HF solution. Subsequently, ~1.0 nm thick Fe films were deposited via electron-beam evaporation. Film thickness in these experiments was evaluated by spectroscopic ellipsometry. The Fe film was then immediately removed using a HCl solution. Following this, the sample was exposed to SiH_4_ at 400 °C, with total gas pressure and exposure duration of 100 Pa and 1800 s, respectively.

## 3. Formation of Ultrathin Si/Fe-Silicide/Si Structures

Figure 1a′–d′ shows atomic force microscopy (AFM) images taken after each process step. The root mean square (RMS) roughness of the SOI substrate surface evaluated from the AFM image was as low as ~0.15 nm, which is almost the same as that before the thinning of the Si layer. AFM images of the surface taken after Fe film deposition and subsequent removal of the Fe film confirmed that the RMS roughness remained the same as before Fe deposition, as shown in Figure 1b′,c′. Furthermore, no significant change in the RMS roughness was observed after SiH_4_ exposure, indicating an extremely flat surface at each process step. To evaluate the chemical bonding features of the surface, we analyzed Si 2p_3/2_ and Fe 2p_3/2_ spectra of the Fe/SOI structure before and after HF etching and subsequent SiH_4_ exposure, as shown in Figure 1e and Figure 1f, respectively, measured by X-ray photoelectron spectroscopy (XPS) using monochromatized Al–K*α* radiation (*hυ* = 1486.6 eV), with the binding energy of each spectrum normalized by the C 1s signal. However, an Fe oxide component in the Fe 2p spectrum after Fe deposition was observed due to air exposure during XPS measurements, which was hardly observed prior to HCl treatment. This result indicates that the unreacted Fe and Fe oxide were completely etched by the HCl treatment. Interestingly, the signal intensity originating from the Si–Si and/or Si–Fe component increased and slightly shifted towards the lower binding energy side after HCl treatment compared to that after Fe film deposition, suggesting that the silicidation reaction of the Si layer surface was induced just after Fe film deposition, despite the absence of heating. In other words, the silicidation reaction proceeds through the deposition of the Fe film onto an atomically flat Si surface, followed by its removal using HCl, without any post-deposition annealing. It is well established that, in silicide systems, the resulting crystal phase is determined by the composition ratio [50]. Therefore, in our process, the silicidation is self-limited due to Fe-supply limited kinetics, which leads to the formation of an abrupt interface. Following SiH_4_ exposure, the Si–Si and/or Si–Fe signal intensities slightly increased while the oxidation of Fe–silicide remains suppressed. Therefore, to evaluate the impact of changes in the chemical bonding features on the sample surface after the SiH_4_ exposure, the Si 2p_3/2_ spectra were also measured at a photoelectron take-off angle of 30°, as indicated in Figure 1g. Angle-resolved analysis of Si2p after SiH_4_ exposure showed a significant increase in the Si oxide component in the surface-sensitive measurement at a photoelectron take-off angle of 30°. These results might be attributed to the SiH_4_ decomposition on the Fe–silicide layer surface even at 400 °C, followed by the growth of a Si layer on the silicide layer surface. In the case of Ni silicide, it is known that the silicidation reaction proceeds at low temperature, as the Ni-adamantane structure serves as a precursor for the formation of NiSi_2_ [51]. Whether a similar mechanism is operative in the Fe silicide system remains an open question, which we aim to address in future studies.

**Figure 2:**
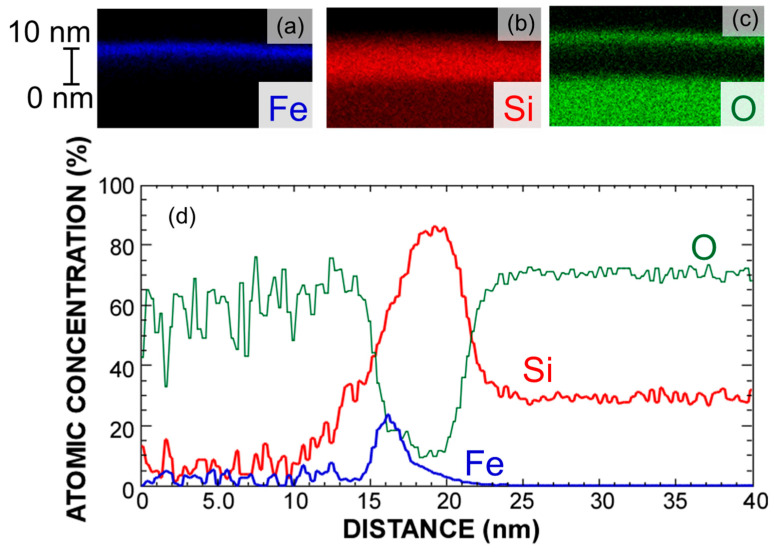
(**a**–**c**) Cross-sectional EDX mapping images and (**d**) cross-sectional profile of sample corresponding to Figure 1d′; in EDX mapping images, blue, red, and green colors correspond to Fe, Si, and O, respectively.

The formation of the ultrathin Fe–silicide layer and Si growth on the silicide layer was confirmed through cross-sectional transmission electron microscopy (TEM) energy-dispersive X-ray spectroscopy (EDX) mapping images, as illustrated in Figure 2a–d. The TEM lamella is prepared by mechanical grinding and polishing followed by Ar ion milling. Despite the Fe film being deposited at room temperature and subsequently etched by HCl, the formation of an ultrathin Fe layer was observed. In contrast, Si signals were detected throughout the sample, irrespective of the film thickness direction. It was confirmed that the top surface of the sample was oxidized, and these results were consistent with those obtained from the XPS analysis. Furthermore, the growth of Si on the Fe surface was confirmed by analyzing the atomic concentration of each element in the film thickness direction, as evaluated from the cross-sectional profile as shown in Figure 2d, indicating the formation of a sandwich structure in which the Fe–silicide layer was sandwiched between the Si layers. It is noteworthy that the atomic concentration ratio of Fe to Si was close to 1:2, suggesting the formation of the FeSi_2_ phase.

## 4. Formation and Light Emission Properties of Fe-Silicide Core/Si-Shell Quantum Dots

**Figure 3:**
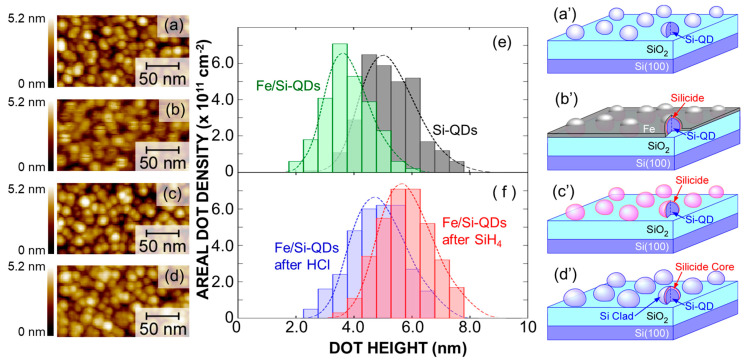
(**a**–**d**) Typical AFM images, (**e**,**f**) dot height distribution evaluated from AFM images, and (**a′**–**d′**) schematic illustrations of (**a**,**a′**) pre-grown Si–QDs, (**b**,**b′**) after Fe film deposition, (**c**,**c′**) after HCl treatment, and (**d**,**d′**) subsequent SiH_4_ exposure, wherein dot height distribution and corresponding curves denote log-normal functions well fitted to measured size distribution.

Based on these results, we applied the same processes as discussed for the SOI substrate, as illustrated in Figure 1, to form a new type of superatom structure, namely, Fe–silicide core/Si–shell QD on an ultrathin SiO_2_ layer. Figure 3a–f shows the results of the AFM topographic images and the dot height distribution evaluated from each AFM image taken after each process step. Firstly, uniformly sized Si–QDs with an areal density as high as ~10^11^ cm^−2^ were self-assembled on a ~3.0 nm thick SiO_2_ layer/p–Si (100) by precisely controlling the low-pressure chemical vapor deposition (LPCVD) using SiH_4_ gas, where the average dot height calculated using a log-normal fitting function was estimated to be ~5.1 nm. After the ~1.0 nm thick Fe film deposition, the surface morphology was slightly smeared, and the average dot height slightly decreased to ~3.9 nm. However, after the HCl treatment, the dot height became the same size, which was ~5.1 nm as that of the as-grown Si–QDs. This result can be explained by complete removal of the unreacted Fe and Fe oxide by the HCl treatment. Subsequent SiH_4_ exposure at 400 °C resulted in a slight increase up to ~5.9 nm with no change in the areal dot density. We also confirmed that no Si deposition occurred on the SiO_2_ surface at 400 °C because the decomposition temperature of SiH_4_ was not reached. Therefore, a slight increase in the average dot height after SiH_4_ exposure can be explained by selective growth of Si on top of the dots. Considering the results of the SOI discussed earlier, these results are expected to form β–FeSi_2_ core/Si–Shell QDs, as schematically illustrated in Figure 3a′–d′. This indicates that the silicidation reaction of the Si–QDs’ surface occurred immediately after Fe film deposition. Subsequently, the unreactive Fe films were etched by the HCl treatment. Followed by SiH_4_ exposure, Si was selectively grown on the Fe silicide surface, acting as an outer cladding.

**Figure 4:**
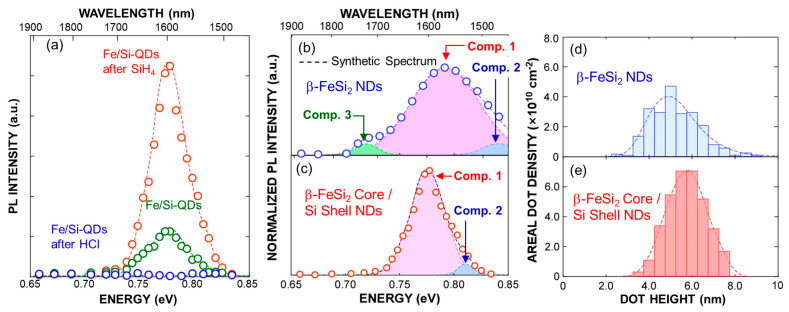
(**a**–**c**) Room temperature PL spectra of (**a**,**c**) NDs are illustrated in Figure 3, and (**b**) β–FeSi_2_–NDs taken under 967 nm light excitations, and (**b**,**c**) their deconvoluted spectra evaluated from spectral analysis using Gaussian curve fitting method, where synthetic spectrum of deconvoluted spectra is also shown as dashed line. Dot height distribution of (**d**) β–FeSi_2_ NDs and (**e**) β–FeSi_2_ core/Si–shell QDs.

**Figure 5:**
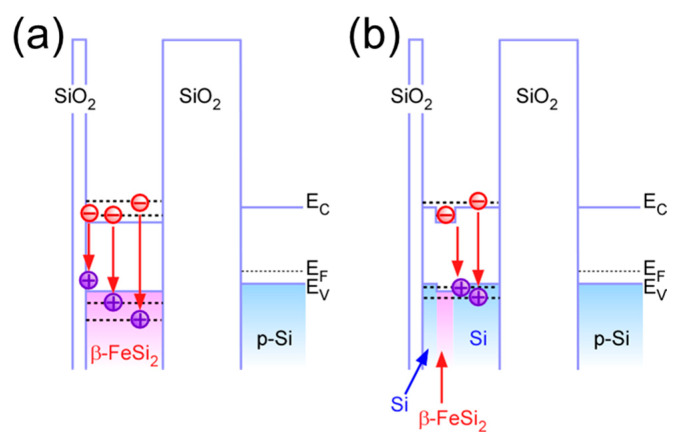
Energy band diagram of (**a**) β–FeSi_2_–NDs and (**b**) β–FeSi_2_ core/Si–shell QDs, illustrating radiative transition corresponding to PL components as shown in Figure 4b,c.

To clarify the crystalline phase of the silicide core, we measured the room temperature PL, using a semiconductor laser with a wavelength of 976 nm at an input power of ~0.33 W/cm^2^ as an excitation source and a cooled InGaAs detector, as shown in Figure 4a. Although a weak PL signal in the range from ~0.73 to 0.83 eV was observed immediately after the Fe deposition, light emission from the sample was hardly detected after HCl treatment. These results can be explained as follows: immediately after the Fe film deposition, as discussed in Figure 2, β–FeSi_2_ layer was formed on the Si–QDs surface despite the oxidation. On the other hand, after HCl treatment, non-radiative recombination centers at the β–FeSi_2_ surface dominated due to the complete etching of the surface oxide. It should be noted that, after SiH_4_ exposure, light emission with no significant change in wavelength was observed, and the PL intensity drastically increased by a factor of five compared to that after Fe film deposition. This result provides clear evidence for the formation of core/shell dots, indicating that the ultrathin Si cap layer was conformally covered with the dots, resulting in the suppression of defects on the β–FeSi_2_ surface. In addition, the PL signal was narrower than that of β–FeSi_2_ NDs with an average height of ~5 nm formed by SiH_4_ exposure of Fe nanodots, as shown in Figure 4b,c. This result can be interpreted in terms of the formation of very uniformly sized core/shell dots compared to the β–FeSi_2_ nanodots, as presented in Figure 4e,f, where the full-width and half-maxim value is ~2.4 nm for the β–FeSi_2_ NDs and ~1.9 nm for the core/shell QDs. To analyze the transition processes, the PL spectrum of the β–FeSi_2_ core/Si–shell QDs was precisely deconvoluted using four Gaussian curves and compared with that of the β–FeSi_2_ NDs, as indicated in Figure 4b,c. As discussed in ref. [48], the PL signal of the β–FeSi_2_ NDs can be deconvoluted into three main components, which peaked at ~0.79 (Component 1), ~0.72 (Component 2), and ~0.84 eV (Component 3) in Figure 4b, in which Comp. 1 can be attributed to the radiative recombination of photogenerated electron–hole pairs through discrete quantized states of β–FeSi_2_ NDs considering the bandgap of single-crystal β–FeSi_2_ bulk. In addition, the radiative transition between the higher-order quantized states in the conduction and valence bands of the β–FeSi_2_ NDs is likely responsible for the higher-energy Comp. 2, as illustrated in Figure 5a. Therefore, Comp. 3 can be attributed to defects in the β–FeSi_2_ NDs and/or the interface between the SiO_2_ and NDs. In contrast, the PL spectrum of the β–FeSi_2_ core/Si–shell QDs was deconvoluted into mainly two components, and the lowest component was hardly detected. However, even though the silicide layer of the core/shell dots is thinner than that of the dots, each PL component is shifted to lower energy compared to the components of the β–FeSi_2_ NDs. In semiconductor nanostructures, the PL peak energy shifts toward the higher energy side while decreasing in size due to quantum confinement effects. Therefore, this energy shift cannot be explained by the size effect of the semiconductor nanostructures. To evaluate the influence of core size in such core/shell structures, a systematic investigation involving the fabrication of Si-QD with different sized β–FeSi_2_ core, as demonstrated in ref. [31], is required. Nevertheless, considering the energy band diagram of the β–FeSi_2_ core/Si–Shell structure, Comp. 1, which has a lower energy emission, might be attributable to radiative recombination between the quantized states in the conduction band of the β–FeSi_2_ core and the valence band of the Si shell, as illustrated in Figure 5b, because electrons are confined in the conduction band of the β–FeSi_2_ core, and holes can be confined in the shallow potential well of the valence band of the Si clad and penetrate the β–FeSi_2_ core for radiative recombination in this system.

The results obtained in this work show that the β–FeSi_2_ core/Si–shell QDs have narrow PL spectra in the region of 0.74–0.84 eV at room temperature, suggesting that the radiative recombination of photo-generated carriers through quantized states of the core is the dominant pathway for the emission from the dots, reflecting the energy band discontinuity between the Si clad and FeSi_2_ core. This means that the β–FeSi_2_ core/Si–shell QDs can be regarded as active elements in optical applications with very few defects at the interface and/or surface, making them one of the most technologically important materials. Furthermore, our samples are highly compatible with the Si–ULSI processing technology. We believe that our findings will pave the way for constructing Si-based photonic processing that exhibits high thermal stability, low power consumption, and room temperature operation.

## 5. Conclusions

We fabricated highly dense β–FeSi_2_ core/Si–shell QDs with an areal density as high as ~10^11^ cm^−2^ on an ultrathin SiO_2_ layer and evaluated their room temperature light emission properties. We confirmed a clear light emission in the near-infrared region at room temperature. The origin of this light emission under weak excitation is considered to be the radiative recombination between the quantized states in the conduction band of the β–FeSi_2_ core and the valence band of the Si shell. Although the β–FeSi₂ core/Si shell QDs fabricated in this study are extremely small in size, their areal density likely results in limited absorption of incident light, leading to low quantum efficiency. Nonetheless, the light emission intensity could be significantly improved by increasing the dot density or by implementing multilayer stacking structures. From a technological point of view, it is quite important that such narrow light emission in the spectral width at room temperature could be achieved using the β–FeSi_2_ core/Si–shell QDs, which have very few defects at the interface between the QD and its surface. The results lead to the development of Si-based light-emitting devices that are highly compatible with Si–ULSI processing, which was found difficult to realize in silicon photonics.

## Data Availability

The original contributions presented in this study are included in the article. Further inquiries can be directed to the corresponding author.
